# The Vertebrate Skeleton

**Published:** 1898-06

**Authors:** 


**The Vertebrate Skeleton.** By SIDNEY H. REYNOLDS. Pp. xvi., 559. Cambridge: At the University Press. 1897.

This is one of the "Cambridge Natural Science Manuals," and is written by a lecturer at University College, Bristol. It consists, briefly, of the following: first come short chapters on the lowest members of the vertebrate phylum, on the various elements of the skeleton, and on the classification of the vertebrate groups. Each group is then taken in turn, an account is given of its general skeletal characters, the skeleton of one or more selected types is described in detail, and lastly the variations throughout the group are pointed out organ by organ. It is, then, like the other books in this series, essentially a practical treatise, intended for use by the student in the laboratory to help him in making his knowledge a real one and as objective as possible. It is a good example of the modern English teaching of biology, which we owe in so large a measure to Professor Huxley.

